# Shifting Priorities in Emergency Medicine: The Rise of Hematological and Hepatological Emergencies and Their Impact on Mortality Rates

**DOI:** 10.7759/cureus.82493

**Published:** 2025-04-18

**Authors:** Prakash Tendulkar, Yasmee Khan, Rajnish Joshi, Mahadev Meena

**Affiliations:** 1 Internal Medicine, All India Institute of Medical Sciences, Bhopal, Bhopal, IND; 2 General Medicine, All India Institute of Medical Sciences, Bhopal, Bhopal, IND

**Keywords:** cardio vascular disease, emergency medicine, hematology, hepatology, mortality

## Abstract

Background

Liver disease is a major global health concern, causing millions of deaths each year. Cirrhosis, a serious complication of liver disease, is a leading cause of disability and healthcare costs. Alcohol abuse is a major contributor to liver diseases, particularly in high-income countries. However, the burden of liver diseases is also rising in developing countries like India due to factors such as lifestyle changes and increasing alcohol consumption. Also, there is an increasing incidence of hematological malignancy globally.

Method

In this study, we analyzed admission and discharge data of patients admitted to a medical ward of a tertiary care hospital in central India. All the patients admitted to the medical ward were included in the study. The records with insufficient patient information were excluded from the study. The data was analyzed from January 2022 to December 2022. The data was analyzed every quarter in terms of counts and percentages. The variables included were the total percentage of admissions and mortality quarterly, the percentage of admissions based on disease type counts on subclassifications of disease category, and the percentage distribution of liver diseases according to gender and cause.

Result

A total of 1083 patients were admitted to the medicine ward from the emergency department, 929 (85.78%) were discharged, 45 (4.15%) were transferred, and 109 died, resulting in a mortality rate of 10.06%. In our study, we found that among non-infectious diseases, cardiovascular diseases still occupy the top spot with 164 (15.14%) admissions and 18 (10.98%) mortality. In comparison, the second position is held by hematological emergencies with 134 (12.37%) admissions and four (2.99%) mortality, while the third highest number of admissions was from hepatological cases with 109 (10.6%) admissions and 19 (17.43%) mortality.

Conclusion

Since data regarding admissions to emergency departments is very limited in India, more data is needed to draw valuable conclusions. However, we want to stress that although cardiovascular diseases are still a top priority in terms of mortality and morbidity, there is now a rising percentage of admissions of hematological and hepatological cases, with hepatological emergencies even having higher mortality.

## Introduction

Liver disease causes around two million deaths each year, representing 4% of all global fatalities (one in every 25 deaths worldwide). About two-thirds of these liver-related deaths occur in men. Alcohol is a major contributor to cirrhosis worldwide, with its incidence being particularly high in high-income countries. Liver disease, including cirrhosis, viral hepatitis, and liver cancer, is responsible for more than two million deaths each year, making up 4% of global fatalities (one in every 25 deaths worldwide). One-third of these liver-related deaths occur in females [1]. Cirrhosis significantly affects global health by generating many disability-adjusted life-years (DALYs). It ranks as the 15th leading cause of DALYs worldwide [2]. Cirrhosis is also a costly disease. In the United States in 2016, liver-related expenses amounted to $32.5 billion (95% CI $27.0-$40.4 billion), with two-thirds of these costs stemming from inpatient or emergency department care. Over the past 20 years, healthcare spending on cirrhosis has risen by 4% annually, mainly due to hospital-based services [3].

Alcohol use disorder (AUD) affects 5.1% of the global population, equating to 283 million people. The highest prevalence rates are found in the European Region, where 14.8% of men and 3.5% of women are affected, and in the Americas, 11.5% of men and 5.1% of women are affected. Notably, AUD is more common in high-income countries and is likely underreported and underdiagnosed in low-income countries [4]. It's important to note that AUD tends to be more common in high-income countries and is likely underreported and underdiagnosed in low-income countries [5].

In India, as in many developing countries, there are limitations in the quality of available epidemiological data on liver disease, including diagnostic precision, clinical phenotyping, consistency in reporting, and the absence of comprehensive electronic databases. Despite these limitations, substantial evidence indicates that liver diseases are increasingly impacting the country's economy and healthcare resources, contributing to premature death and disability. The ongoing cultural and lifestyle transition in India, characterized by the adoption of a Western diet, sedentary habits, and a shift in societal attitudes towards alcohol, is fostering conditions for a rapidly changing spectrum of liver diseases [6]. This shift includes the growing significance of alcoholic liver disease (ALD) and nonalcoholic fatty liver disease (NAFLD) as leading causes of liver disease, surpassing viral causes [7]. In 2016, various chronic liver diseases (CLDs) and cirrhosis were responsible for 2.1% of all deaths in India [8].

One challenge that delays early treatment and strains healthcare resources is that chronic liver diseases (CLDs) in India often manifest late in the clinical course, frequently after decompensation has occurred [6]. Although no data is available on hematological emergency admissions, it has been reported that the number of new cases of hematologic malignancies has been rising since 1990, reaching 1,343.85 thousand in 2019. However, the Age-Standardized Death Rate (ASDR) for these malignancies has been decreasing [9].

## Materials and methods

Study design and participants

The current study was performed at the All India Institute of Medical Sciences, Bhopal, A tertiary care hospital in Central India. Patients with a medical illness are admitted to the medical wards through outpatient or emergency services. During the study period, patients were admitted to three medical units, with a prespecified equal number of admission days (two weekdays and Sundays by rotation). Thus, on average, each unit has 10 admission days per month. The treating physicians in each unit make the decision to admit a patient based on the perceived severity of illness. The medical units offer admission to patients with diseases regardless of the affected organ system. Care of some of these patients is subsequently transferred to more specialized units.

We designed a retrospective chart review of the patients admitted to medical wards from emergency services for a period of one calendar year (January to December 2022). The study design was approved by the Institutional Human Ethics Committee at All India Institute of Medical Sciences, Bhopal (LOP number AIIMSBPL/IHECSR/NOV-2024/18).

Data source 

We considered patient records as the data source for the study. These records include an initial admission slip, daily progress notes by the treating doctors and nurses, and monitoring and drug administration records. Every patient record has a summary prepared by the treating physicians at the time of discharge or death. This summary includes demographic details, duration of stay, diagnosis, and outcome of the current hospitalization. Each patient is identified by a unique case record number that remains unchanged for subsequent hospitalizations.

Inclusion and exclusion criteria

We included all admission records of patients admitted to one of the three treating medical units from the hospital emergency services. This ensured that we were able to consistently sample such admission records for 10 days in a month. The records were sampled for admission dates between 1st January and 31st December 2022. Records from preceding years were not included in the study, as in the years 2020 and 2021, there were periods when the hospital exclusively admitted patients with COVID-19 infection, which had an overwhelming impact on the overall disease spectrum. We also excluded those records that had an admission duration of less than 24 hours. This exclusion was applied because such records have insufficient information about the patient’s disease condition.

Methodology 

Each record was evaluated by two members of the study team (YK and PT). We abstracted the information about demography (age and gender), hospitalization (date of admission, duration of stay, and discharge outcome), and diagnosis at the time of discharge or death. The study team members then evaluated the final diagnosis, based on the details mentioned in the progress notes, to identify the primary reason for hospital admission. Only one primary diagnosis was considered for each patient, and disagreements were resolved by consensus. Each diagnosis was then classified into infectious and non-infectious categories. The diagnoses in the non-infectious category were then subclassified as vascular, hepatic, renal, pulmonary, autoimmune, neurology, hematologic, endocrine, malignancy, and poisonings. Those who could not be classified in the above subgroups were miscellaneous.

Analysis

The data was analyzed quarterly in terms of counts and percentages. The variables included were the total percentage of admissions and mortality on a quarterly basis, the percentage of admissions on the basis of disease type counts on subclassifications of disease category, and the percentage distribution of liver diseases according to gender and cause.

## Results

Of the 1083 patients admitted to the medicine ward from the emergency department, 929 (85.78%) were discharged, 45 (4.15%) were transferred, and 109 died, resulting in a mortality rate of 10.06% (Table 1).

**Table 1:** Total admissions in each quarter and percent mortality

Quarter	Total	Death	Discharged	Transferred
Jan-March 2022	94 (8.67%)	9 (8.25%)	80 (8.61%)	5 (11.11%)
April-June 2022	281 (25.94%)	32 (29.35%)	237 (25.51%)	12 (26.66%)
July-Sept 2022	403 (37.21%)	36 (33.02%)	353 (37.99%)	14 (31.11%)
Oct-Dec 2022	305 (28.16%)	32 (29.35%)	259 (27.87%)	14 (31.11%)
Total	1083	109	929	45

In the non-infectious diseases category, the highest number of admissions was for cardiovascular cases, with 164 (15.14%) patients. Of these, 139 (84.75%) were discharged, 18 (10.98%) died, and seven (4.26%) were transferred. This category accounted for 15.14% of all admissions and had a mortality rate of 10.98%, with a mean patient age of 57.41 years (Table 2).

**Table 2:** Total admissions by disease category and percent mortality in non infectious diseases

Disease	Total	Death	Discharged	Transferred	Mean Age (years)
Vascular	164 (15.14%)	18 (16.51%)	139 (14.96%)	7 (15.55%)	57.41
Hematology	134 (12.37%)	4 (3.66%)	125 (13.45%)	5 (11.11%)	36.74
Liver	109 (10.06%)	19 (17.43%)	89 (9.58%)	1 (2.22%)	47.09
Endocrine	62 (5.72%)	0	61 (6.56%)	1 (2.22%)	51.20
Poisoning	46 (4.24%)	3 (2.75%)	41 (4.41%)	2 (4.44%)	29.14
Neurology	35 (3.23%)	1 (0.91%)	30 (3.22%)	4 (8.88%)	46.08
Pulmonary	34 (3.13%)	4 (3.66%)	28 (3.01%)	2 (4.44%)	61.71
Cancer	23 (2.12%)	4 (3.66%)	16 (1.72%)	3 (6.66%)	48.25
Renal	22 (2.03 %)	4 (3.66 %)	17 (1.82 %)	1 (2.22 %)	57.25
Pancreas	20 (1.84%)	2 (1.83%)	18 (1.93%)	0	38.67
Autoimmune	19 (1.75%)	4 (3.66%)	15 (1.61%)	0	39.50
Miscellaneous	42 (3.87%)	0	39 (4.19%)	3 (6.66%)	52.67
Infectious	373 (34.44%)	46 (42.20%)	311 (33.47%)	16 (35.55%)	46.82
Grand Total	1083	109	929	45	45.54

The next largest group was hematological cases, with 134 (12.37%) admissions. Of these, 125 (93.28%) were discharged, four (2.98 %) died, and five (3.73%) were transferred. Hematological cases comprised 12.37% of total admissions, with a mortality rate of 2.98% and a mean age of 36.74 years. In the liver disease category, there were 109 (10.06%) admissions, 89 (81.65%) discharges, 19 (17.43%) deaths, and one (0.91%) transfer with a mean age of 47.09 years. Endocrine diseases followed with 62 (05.72%) admissions, 61 (98.38%) discharges, and one (1.61%) transfer. The poisoning category included 46 (4.24%) admissions, with 41 (89.13%) discharges, three (6.52%) deaths, and percentages of admissions and mortality at 4.24% and 6.52%, respectively, and a mean age of 29.14 years.

The neurology group had 35 admissions (3.23%), 30 (85.71%) discharges, and four (11.42%) transfers, with a mortality rate of one (2.85%) and a mean age of 46.08 years. The pulmonary group had 34 admissions (3.13%), 28 (82.35%) discharges, two (5.88%) transfers, and a mortality of 4 (11.76%), with a mean age of 61.71 years. In the malignancy and renal groups, there were 23 (2.12%) and 22 (2.03%) admissions, respectively. Both groups had four deaths, with three (13.04%) transfers in the malignancy group and one (4.54%) in the renal group. The mortality rates were 17.39% for malignancy and 18.18% for renal cases, with mean ages of 48.25 and 57.25 years, respectively. The autoimmune group had 19 (01.75%) admissions, with 15 (78.94%) discharges and four (21.05%) deaths, an admission percentage of 1.75%, a mortality rate of 21.05%, and a mean age of 39.50 years. Finally, there was a miscellaneous category, which included 42 (03.87%) admissions, 39 (92.85%) discharges, and three (7.14%) transfers.

In terms of disease distribution within these high-burden categories, the cardiovascular group had the highest number of admissions for ischemic conditions, followed by heart failure and stroke. In the haematology group, anaemia was the most common reason for admission, followed by malignancies and hemoglobinopathies. The majority of hepatology admissions were due to cirrhosis, followed by hepatitis.

In the cardiovascular category, the highest number of admissions included ischemic heart disease, 56 (34.14%), followed by stroke and heart failure with 39 (23.78%) each, valvular heart disease included 19 (11.58%), pericardial one (0.60%), congenital two (1.21%) and other vascular causes such as pulmonary embolism, deep vein thrombosis, etc. were five (3.04%) (Table 3).

**Table 3:** Cardiovascular cases distribution

Disease	Total, N (%)
Aortic/Congenital	2 (1.21%)
Heart Failure	39 (23.78%)
Hypertension	3 (1.82%)
Ischemic	56 (34.14%)
Pericardial	1 (0.60%)
Stroke	39 (23.78%)
Valvular	19 (11.58%)
Other	5 (3.04%)
Total	164

In hepatology cases, out of 109 (10.06%) admissions, 100 (91.74%) patients were admitted due to cirrhosis-related complications, while nine (8.25%) patients were admitted due to fulminant hepatic failure. In the cirrhosis group, 45 (45%) admissions were due to alcoholic cirrhosis, five (11.11%) died, and 40 (88.88%) were discharged. Fourteen (14%) patients were admitted due to hepatitis B-related cirrhosis, five (35.71%) died, and nine (64.28%) were discharged. In the hepatitis C group, there were three (3%) admissions with three discharges. In the autoimmune and cryptogenic groups, there was one admission each with subsequent discharge. There was a substantial group of patients with cirrhosis due to unknown etiology, with 36 (36%) admissions, 33 (91.66%) discharges, and three (8.33%) mortality (Table 4).

**Table 4:** Cirrhosis cases distribution Hep B- Hepatitis B; Hep C- Hepatitis C

Disease	Total	Discharge	Death
Alcohol	45	40 (88.88%)	5 (11.11%)
Hep B	14	9 (64.28%)	5 (35.71%)
Hep C	3	3 (100%)	0
Autoimmune	1	1 (100%)	0
Cryptogenic	1	1 (100%)	0
Unknown Etiology	36	33 (91.66%)	3 (8.33%)

While in hematology, most of the admissions were due to severe anemia that is 64 (47.76 %) cases, malignancies 27 (20.14 %) cases, followed by hemoglobinopathy 20 (14.92 %) cases, pancytopenia 12 (8.95%), immune thrombocytopenic purpura (ITP) seven (5.22 %) and aplastic anemia four (2.98 %) cases (Table 5).

**Table 5:** Hematology cases distribution ITP- Immune Thrombocytopenic Purpura

Disease	Total, N (%)
Anemia	64 (47.76%)
Hemoglobinopathy	20 (14.92%)
ITP	7 (5.22%)
other Malignancy	27 (20.14%)
Pancytopenia	12 (8.95%)
Aplastic Anemia	4 (2.98%)
Total	134

## Discussion

Our study tried to look for the current trend of emergency hospital admissions. We had seen that apart from cardiovascular emergencies, hematological and hepatological emergencies are rising. There has been minimal data regarding emergency medicine admissions worldwide, as emergency medicine is a new branch. Still, some previous studies suggest that emergency admissions are the major contributor to hospital burden and health care costs. One multicenter analysis done by Numico et al. (2020) showed that almost 80.5% of the urgent admissions were responsible for longer hospital stays, leading to higher healthcare costs [9].

In our study, we also found that among non-infectious diseases, cardiovascular diseases occupy the top spot with 164 (15.14%) admissions and 18 (10.98%) mortality. In comparison, the second position was held by hematological emergencies with 134 (12.37%) admissions and four (2.98%) mortality, while the third highest number of admissions were from hepatological cases with 109 (10.06%) admissions and 19 (17.43%) mortality. As we can see, hepatology cases have replaced respiratory emergencies, which now have reduced admissions to up to 34 (3.13%) but a mortality of four (11.76%). Poisoning cases have an admission number of 46 (4.24%) and a mortality of three (6.52%), in fifth position, and next in line are neurological cases with an admission number of 35 (3.23%).

If we look at the data, although cardiovascular diseases still are the top priority among emergency cases, even during the COVID-19 pandemic, the cardiovascular disease burden was very high and was one of the leading causes of emergency hospital admissions [10]. However, what has changed is that hematological and hepatological cases are now the next priority, not respiratory cases. A similar drop in respiratory cases was seen in one of the retrospective ecological studies done in Korea between January 2016 and January 2020, where they found a drop in the number of pneumonia, influenza, chronic obstructive pulmonary disease (COPD) cases, and asthma cases [11]. According to Zhang et al., the number of new cases of hematologic malignancies has been rising globally since 1990. However, the Age-Standardized Death Rate (ASDR) for these malignancies has been decreasing [12].

The pathophysiology of the hematopoietic system is complex, and its blend of cells, plasma, and plasma proteins, which can be affected by disorders stemming from insufficient or excessive blood cell production, the breakdown of existing blood cells, or functional abnormalities in blood cells or their components. The specific symptoms of these disorders vary depending on the affected blood cell lineage, the tissues involved, and the nature of the dysfunction [13]. In our study, among the hematological cases, the largest group of patients was anemia patients, that are 47.76%. A similar trend was seen in one of the large ecological studies done in England and Wales, where they reviewed data from 1999 to 2019 and found there is a 137.9% rise in admissions for blood and blood-forming organs and certain disorders involving the immune mechanism (B and ID) and anemia being the most common presentation [14].

In hepatological cases, the admission was 109 (10.06%), while the mortality percentage was 19 (17.43%). According to Garg et al., in all emergency admissions, coronary artery disease was 9.39%, stroke 7.71%, alcoholic liver disease 6.93%, and chronic obstructive lung disease 3.90% [15]. This is similar to our study, in which cardiovascular patients had 15.14% admissions, hepatological admissions 10.06%, and respiratory diseases 3.13%. But if we take a look at the mortality percentage of 17.43% in hepatological admissions, it indicates that it is higher than that of cardiovascular diseases, which have a mortality percentage of 10.98%. If we look at pulmonary admissions, they still have a high mortality percentage of 11.76% despite a low admissions percentage.

In hematological admissions, we can see that despite the high admission percentage, their mortality percentage was low at 2.98%; this may be due to the fact that most of the admissions comprised anemia and hemoglobinopathy cases, which have low mortality. The prevalence of hematological cases in the form of anemia is already very high in the country due to multiple factors like nutritional deficiency (iron and vitamin B12 deficiency) and genetic factors (Thalassemia and sickle cell disease) [16].

If we look at hepatological admissions, most admissions were due to complications of cirrhosis of the liver, which is 100 (91.7%). Acutely decompensated cirrhosis involves the emergence of cirrhosis-related issues like ascites, hepatic encephalopathy, variceal bleeding, or bacterial infections, with 30% of patients progressing to extrahepatic organ failure and acute-on-chronic liver failure (ACLF). The 90-day mortality remains high, from 14% in acute decompensation to 50% in ACLF, largely due to a lack of effective treatments to halt disease progression [17,18]. 

In cirrhosis cases, most patients belonged to alcohol-induced cirrhosis of the liver, which is 45%. This may be because in India, like most developing countries, there has been a change in the standard of living and a change in the attitude of society towards alcohol consumption, which has significantly increased in the last 20 years. As we all know, data on liver diseases is limited in our country, but according to Mondal et al alcohol-induced cirrhosis cases occur at a frequency of 10.9%-31.9% in various regions of India, and the prevalence of Hepatitis B and C have been on the decline due introduction of National viral hepatitis control program since 2018 which is similar to our study where admission due to Hepatitis B and C related cirrhosis of the liver were less as compared to alcohol-related cirrhosis admissions [7]. A similar trend is seen in a Lancet study published in 2023. The study highlighted the increasing burden of liver diseases in the UK over the last decade [19].

Limitations

As this is just a descriptive analysis of patient data for one year, only more annual data analyses are needed to draw better conclusions. Furthermore, epidemiological and statistically significant studies are needed in this field.

## Conclusions

Our study revealed a shifting landscape of disease burden in emergency admissions. We want to stress that although cardiovascular diseases are still a top priority in terms of mortality and morbidity, there is now a rising percentage of admissions of hematological and hepatological cases, with hepatological emergencies even having higher mortality, leading to major drivers of emergency care utilization. Since data regarding admissions in emergency departments is very limited in our country, Further research is crucial to understand the underlying causes and to develop targeted interventions to address this evolving pattern of emergency admissions and improve outcomes for patients.
